# Cerclage performance analysis – a biomechanical comparison of different techniques and materials

**DOI:** 10.1186/s12891-022-05983-6

**Published:** 2022-12-01

**Authors:** L. M. Hägerich, F. G. E. Dyrna, J. C. Katthagen, P. A. Michel, L. F. Heilmann, A. Frank, M. J. Raschke, B. Schliemann, O. Riesenbeck

**Affiliations:** Department of Trauma, Hand and Reconstructive Surgery, University Hospital Münster, Albert-Schweitzer-Campus 1, Building W1, 48149 Münster, Germany

**Keywords:** Long bone fracture, Steel wire cerclage, Fibertape, Fracture fixation, Biomechanical properties

## Abstract

**Background:**

Wire cerclages play a fundamental role in fracture fixation. With an increasing variety of designs being commercially available the question arises which cerclage should be used.

This study investigates the biomechanical properties of metallic and non-metallic cerclages and their different application-types. Furthermore, potential influence of muscular interposition between bone and cerclage constructs was tested.

**Methods:**

Samples of the following four different cerclage types were tested on 3D printed models of human humeri as well as on human cadaveric humeri with and without muscular interposition:

Titanium Cable Cerclage (CC), Steel Wire Cerclage (SWC), Suture Tape (ST), Suture Tape Cerclage (STC) with both single- (sSTC) and double-loop application (dSTC). A preinstalled self-locking mechanism secured by the provided tensioner in the STCs being the main difference to the STs.

Cyclic loading was performed to 1 kN and then linearly to a maximum load of 3 kN.

Statistical analysis was performed using either one-way ANOVA and post-hoc Tukey or Kruskal–Wallis and post-hoc Dunn test depending on normalization of data (*p* < 0.05).

**Results:**

Whilst all cerclage options could withstand high loads during failure testing, only within the CC and dSTC group, all samples reached the maximal testing load of 3000 N without any failure. The SWC reached 2977.5 ± 63.6 N, the ST 1970.8 ± 145.9 N, and the sSTC 1617.0 ± 341.6 N on average.

Neither muscular interposition nor bone quality showed to have a negative influence on the biomechanical properties of the cerclage constructs, presenting no significant differences.

**Conclusion:**

All tested cerclage constructs produce reliable stability but differ in their resulting compression forces, in a simplified fracture model. Therefore, non-metallic cerclage alternatives can provide similar stability with less compression and stiffness to metallic cable constructs, but they may offer several advantages and could possibly provide future benefits. Especially, by offering more elasticity without losing overall stability, may offer a biologic benefit. Installing any cerclage constructs should be performed carefully, especially if poor bone quality is present, as the tightening process leads to high forces on the construct.

**Supplementary Information:**

The online version contains supplementary material available at 10.1186/s12891-022-05983-6.

## Background

The cerclage as a circumferential stabilizer displays a fundamental role in fracture treatment. It can be helpful during fragment reduction and may add significant stability to the final construct. However, some studies have shown an increased risk of non-union by restricting periosteal blood supply [1].

Primarily, steel wire cerclages were introduced in the eighteenth century as they were easy to use, cheap and robust [2]. But over time, more and more downsides and problems of the additional metallic wires were reported [3, 4].

With the evolution of high-strength sutures and tapes, new alternative materials are available, offering potential benefits. Additionally, suture-based constructs are radiolucent, have a wide field of use that is not limited to fracture care, are sterile on demand and eliminate the risk of metallosis or metal-related allergies. Without those sharp ends, the steel wire cerclage has, they are safer for the surgeon to use. Further, the potential cause of soft tissue irritation for the patient is reduced and they are easier to revise [5].

This study aimed to compare commercially available metallic and non-metallic cerclages in a simplified fracture model. Additionally, the validity and reproducibility of tensioning devices on the designated compression force were evaluated. To create a clinically relevant scenario, the influence of muscular interposition between bone and cerclage constructs was designed and tested.

It was hypothesized that non-metallic constructs would deliver comparable biomechanical results to metallic cerclage constructs. Further it was hypothesized that, muscular interposition would not limit the stability of either construct.

## Methods

### Tested cerclage materials and techniques

Four commercially available cerclage materials were utilized for the comparison. The installation was performed under the best visual conditions. The cerclages were installed by one single surgeon with appropriate level of training and clinical experience. Each cerclage was only used once, and no further manipulation or adjustment after the installation was allowed. The maximum compression force of each construct was documented before testing.

### Titanium cable cerclage (CC)

**Fig. 1:**
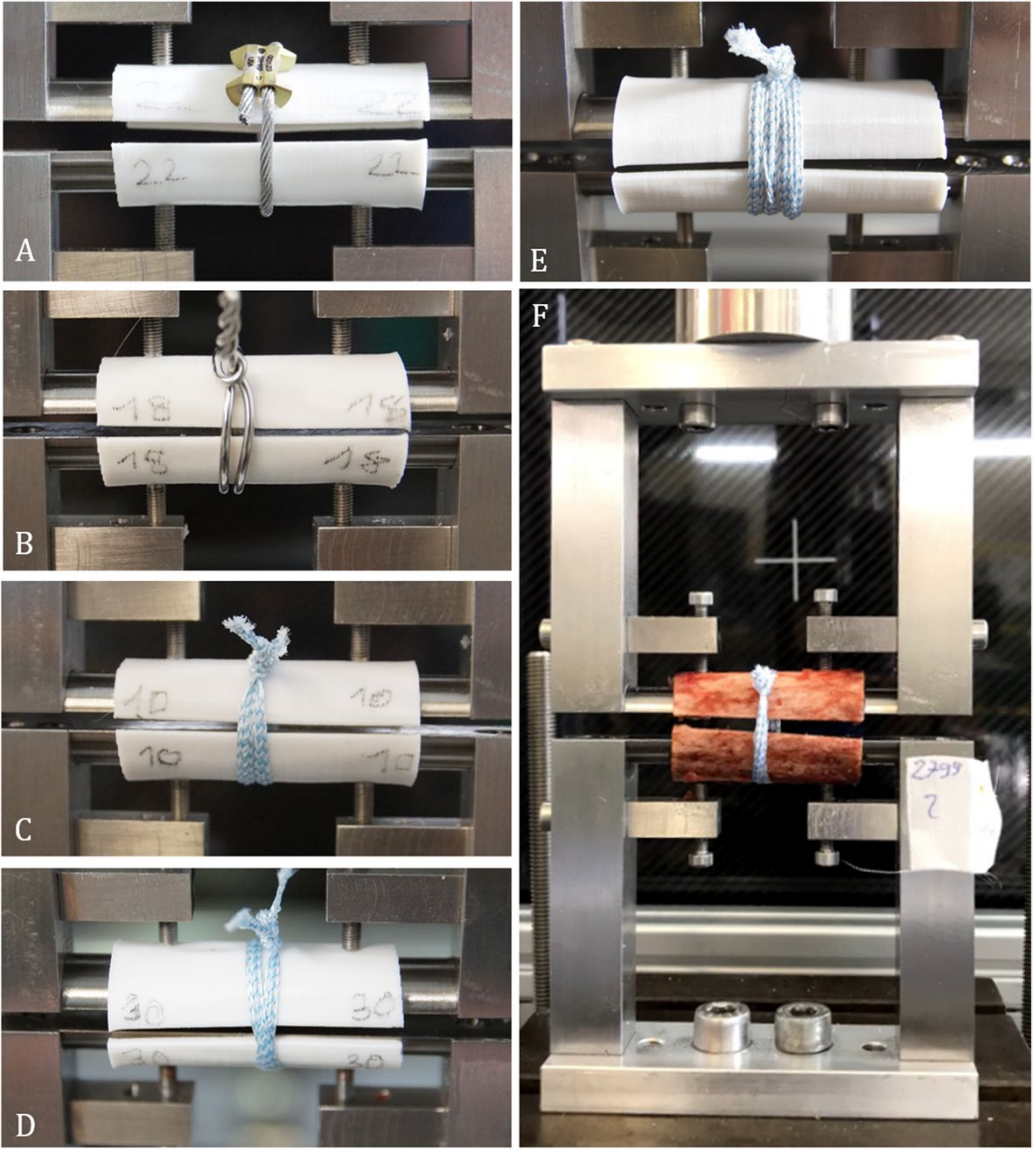
**A**-**E** Displaying all installed cerclage Options on the 3D printed half shell constructs. **F** Showing the complete biomechanical test set-up with the custom designed grip to individually adjust and place the half shell constructs perfectly into the servohydraulic testing machine

The Cerclage Cable Ø 1.7 mm, (DePuy Synthes, Synthes, Oberdorf, Switzerland) was manually positioned once around the half shell, and the crimp was placed as recommended in the manual. A matching tension device was introduced, and the cerclage was tensioned to 40.0 kg on the testing device scale according to manufacture guideline. The crimp was locked, the cerclage trimmed, and the tensioning device removed. (Fig. 1A).

### Steel wire cerclage (SWC)

A Ø 1.5 mm steel wire (Coil w/Cerclage wire Ø 1.5 mm, DePuy Synthes, Synthes, Oberdorf, Switzerland) was placed around the humerus twice, as previous literature has evaluated a comparable strength of double steel wire compared to single cable cerclage [6]. The ends were gripped with pliers and twisted clockwise under continuous tension for six turns [7]. (Fig. 1B).

### Suture tape (ST)

The high-strength suture tape (FiberTape, Arthrex GmbH, Munich, Germany) was twisted around the bone shell twice and knotted using seven alternating half hitches with a focus on maximum possible compression force. (Fig. 1C).

### Suture tape cerclage (STC)

In contrast to the simple Suture Tape, this version entails a preinstalled self-locking mechanism similar to a Chinese finger trap (FiberTape Cerclage, Arthrex GmbH, Munich, Germany). The test setup was performed in two different variations. The first setup contained a single loop (sSTC), resulting in a two-strand construct (Fig. 1D). This is not in accordance with the manufacture's recommendations but did reflect a more comparable situation to the other constructs. For the second version, in accordance with the manufacturer's recommendation, a double loop (dSTC) was performed, creating a four-strand construct. All STCs were tensioned as described in the manual, using the provided tensioner up to the value of 60 (representing 60 lbs = 27.2 kg) and secured by two alternating half hitches. (Fig. 1E).

### Specimen preparation & fixation

#### 3D printed model (*N* = 8 of each group)

To create identical samples for objective material testing, a bone model was printed. Therefore, 40 mm long half-shells were designed by using a CT scan of a matching proximal humerus with an inner cortical diameter of 15 mm as a blueprint. The printed walls of these objects were filled entirely with ABS- filaments (UFP Germany GmbH, Kamp-Lintfort) in a Flashforge Creator Pro 3D printer (Flashforge, Waldshut-Tiengen, Germany), to simulate a cortical character. The cortical thickness in the 3D printed half shells was set to 1 mm.

#### Cadaveric samples (*N* = 8 of each group)

Seven human cadaveric humeri, mean age 79.1 ± 4 years (2F/5 M), were obtained and underwent QCT for bone mineral density measurements. A 1 cm^2^ region within the proximal third of the humeri were measured and yielded an average of 115.1 ± 33,3 mg/cm^3^. The muscular tissue adhering to the bones was dissected. Deltoid muscles were kept separately to simulate muscular interposition in a later step. The humeri were cut into 35 mm long pieces, starting underneath the calcar region. To create those beforementioned half-shells, the bone pieces were sawed open longitudinally, and the remaining bone marrow was removed, resulting in matching cortical half-shells. The cortical thickness of the human samples was not artificially unified by reaming the bone to a certain thickness. As the half shells had to properly fit onto the custom-made rig, the diameter of the medullary cavity was the limiting factor to the use of the samples. We got up to three samples out of a single specimen due to size differences. However, bone fractures where never the limiting factor regarding cerclage performance.

#### Muscular interposition (*N* = 8 of each group)

**Fig. 2:**
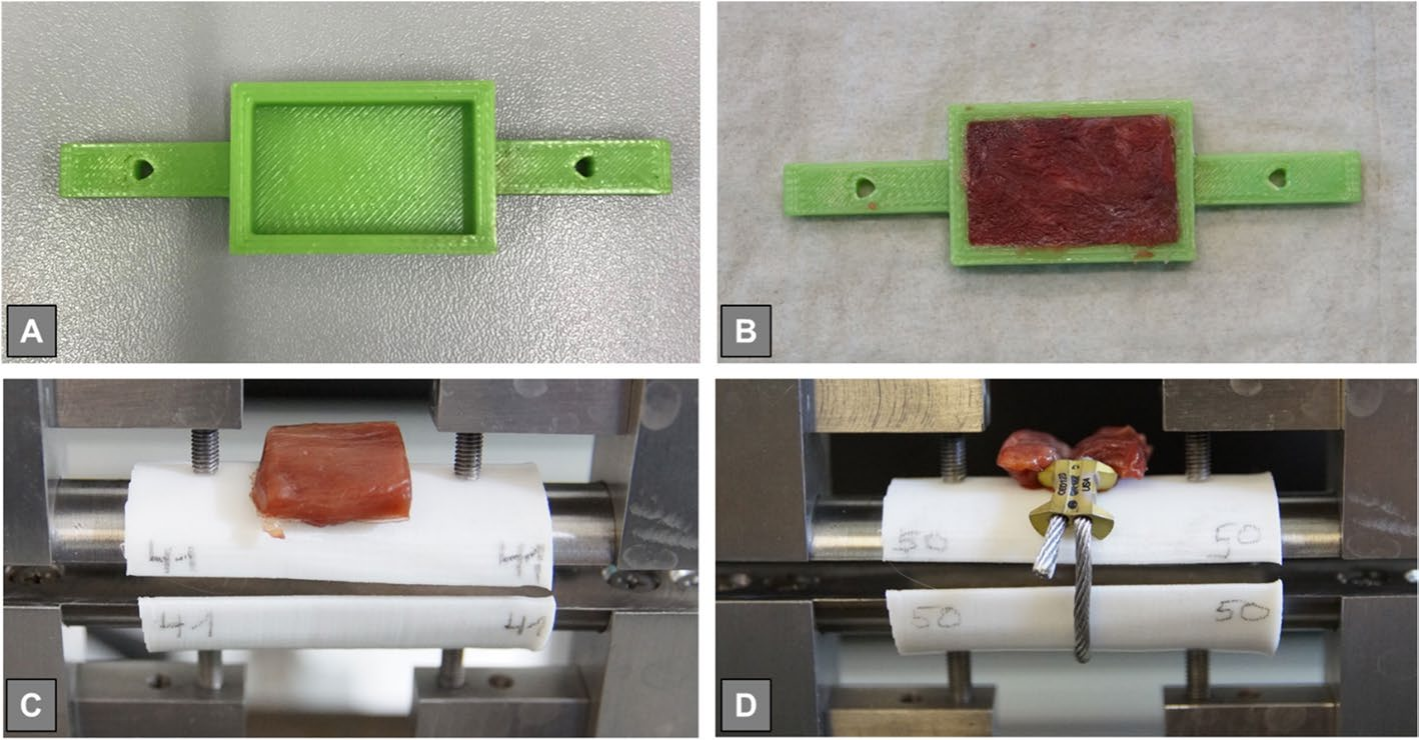
Demonstrating the procedure to create comparable muscle interposition grafts (**A**-**B**) and the installation of a cerclage on top (**C**-**D**)

To simulate the clinical effect of soft-tissue interposition, a muscular graft was designed and placed onto the 3D-printed bone shells. Grafts were created by filling up a 25 × 15x4 mm printed box with a deltoid muscle flap (Fig. 2). To control the height of the flap, it was frozen within the box and could then be sharply resected along the edges of the box without any manual pressure. The thickness of 4 mm was chosen to reflect a realistic clinical scenario. Muscular grafts were implemented with fibers being in line with the simulated half-shell fracture structure.

### Testing sequence

**Fig. 3:**
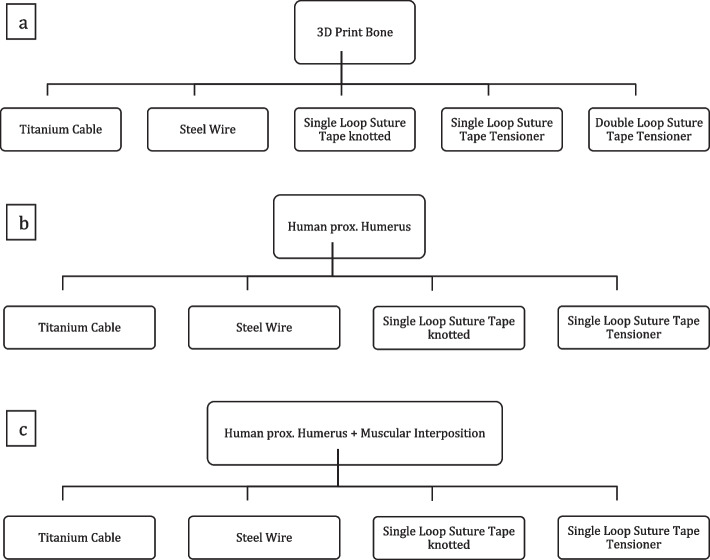
**a**-**c** Displaying the testing sequence of used material and installed cerclage techniques

The biomechanical testing was performed in three setups shown in Fig. 3a-c. First, the 3D printed bone model was created to rule out material differences as a variable to compare isolated cerclage performance. Secondly, human cadaveric proximal humeri were utilized to simulate adequate bony anatomy and stability for clinical validation of the results. Finally, an additional muscular interposition was simulated by interposing a deltoid muscle flap on the backside (out of view area) of the human bone samples. No sample was reused during testing.

### Cerclage manipulation

In a clinical scenario, the influence of cerclage manipulation after final implantation was evaluated as a potential cause for loss of reduction. Especially the wire cerclages are known to rely on perfect closure and lock position. However, it is not uncommon to manipulate or reposition the twisted cerclage lock during surgery. Therefore, the influence on compression force and final load to failure for wire cerclages and suture tape cerclages (*N* = 6 per group) were compared using the 3D printed half-shells. The steel wire and the suture tape constructs were chosen as their installation is performed without any guiding device and therefore thought to be more influential by manual manipulation. The intraoperative manipulation was simulated by flipping the twisted ends or knot stacks 3 times from one side to the other using pliers. The constructs were tested afterward according to the same study protocol.

### Testing protocol

For biomechanical testing, half-shells were placed into a servohydraulic testing machine.

(Instron, Model 8874, Instron GmbH, Pfungstadt, Germany). To secure the shells, reproducibly, a custom rig was designed. (shown in Fig. 1F) The shells were fixed by screws, and rotation was eliminated. The bottom part of the rig was rigidly fixed, whereas the upper part was connected to a 10 kN load cell. All parts could be aligned freely to match the half-shells perfectly. Due to the two-part grip design, a designated fracture gap of 1 mm was created between the shells. Hence the compression force of each construct could be evaluated additional to the overall construct stability. The following parameters were collected: (1) compression force at time point zero, (2) force difference between installation and final construct, (3) maximum force, (4) required force to elongate the construct 3 mm, (5) overall construct displacement after cyclic loading. A 3 mm elongation was set as a clinical failure of the construct according to Renner et al. as no further stabilizing effect will be present [8].

The following test sequence was performed similar to existing literature [7]:

1. Installation of cerclage construct and collection of compression force
2. Preconditioning with 10 cycles (10–100 N)
3. Cyclic ramped test interval to mimic early post-operative motion starting at 25 N and 5 N increments for each cycle until 1 kN at 2 Hz.
4. Followed by a linear load to failure testing at 10 mm/min until a maximum of 3 kN in order not to damage the grip.

### Statistics

All data was generated using individual MATLAB script (MATLAB R2018b, MathWorks, Natick, USA). GraphPad PRISM (Version 8, GraphPad Software, San Diego, USA) was utilized for all statistic tests. Test were performed within the tested groups shown in Fig. 3. If normal distribution was present, a one-way ANOVA with a post hoc Bonferroni correction was performed. For non-normalized data, a Kruskal Wallis test was applied. Furthermore, p-values between groups were compared and adjusted by either Dunn or Tukey. A *p*-value less than 0.05 was considered statistically significant.

## Results

### Mechanical properties of each cerclage on 3D printed bone shells

**Fig. 4:**
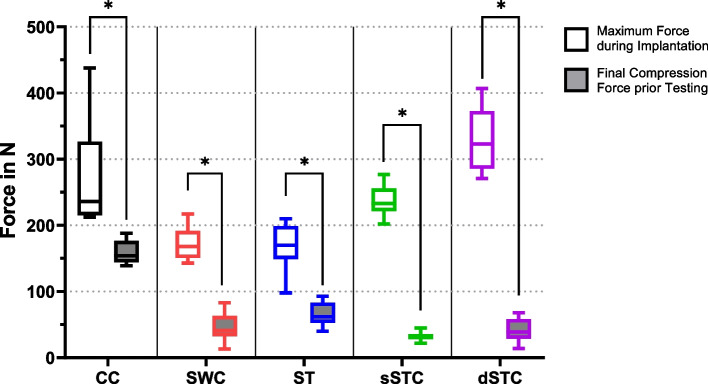
Is showing the maximum compression force during cerclage installation and the final construct compression force (*p < 0.001)

One evaluated quality of the constructs was their power to create a compression force onto the simulated fracture site by pressing the half-shells together. In this study, the cable cerclage (CC) reproducibly reached the highest forces with 159.0 ± 17.7 N. This was significantly higher than the other constructs with steel wire (SWC) 64.3 ± 21.9 N, suture tape manual knotted (ST) 65.8 ± 17.8 N, single suture tape with tensioning device (sSTC) 32.5 ± 6.9 N, and double suture tape with tensioning device (dSTC) 41.0 ± 17.8 N with *p* < 0.001. The results were independent of the used shell material. Secondly, the force difference between installation and final construct was evaluated. The test setup was able to show that each of the cerclage systems loses a significant amount of tension or causes a significant amount of stress during implantation steps and final fixation with maximum values of CC 270.3 ± 81.3 N, SWC 171.8 ± 24.9 N, ST 168.1 ± 37.4 N, sSTC 237.9 ± 23.7 N, dSTC 330.4 ± 49.2 N, *p* < 0.05 for all constructs. Those results are displayed in Fig. 4.

All cerclage options were able to exceed high loads during failure testing. However, within the CC or dSTC group, all sample reached the final values of 3000 N, which was set as maximum testing load without any failure. While the SWC reached 2977.5 ± 63.6 N, the ST 1970.8 ± 145.9 N, and the sSTC 1617.0 ± 341.6 N on average.

To further evaluate the material differences in stiffness, the needed force to reach a displacement of 3 mm during ramped cyclic loading up to 1000 N was evaluated for each cerclage setup. With an average displacement of only 2.7 ± 0.9 mm, the cable cerclage (CC) was the stiffest construct with 7/8 samples not reaching the 3 mm barrier at 1000 N. The SWC group elongated over 3 mm at 830 ± 80 N, and the ST group at 670 ± 220 N. The sSTC represented the most elastic construct compared to all others resisting 370 ± 80 N before reaching the 3 mm (*p* < 0.001). However, by using the dSTC, the construct stiffness could also be doubled with averaging 650 ± 180 N prior to reach the 3 mm displacement benchmark.

### Influence of human samples and muscular interposition

Neither the individual shape of the samples, nor the bone quality, nor muscular interposition showed to have a negative influence on the biomechanical properties of the cerclage constructs, presenting no significant differences for the compression forces.

### Influence of manipulation during the surgical procedure

**Fig. 5:**
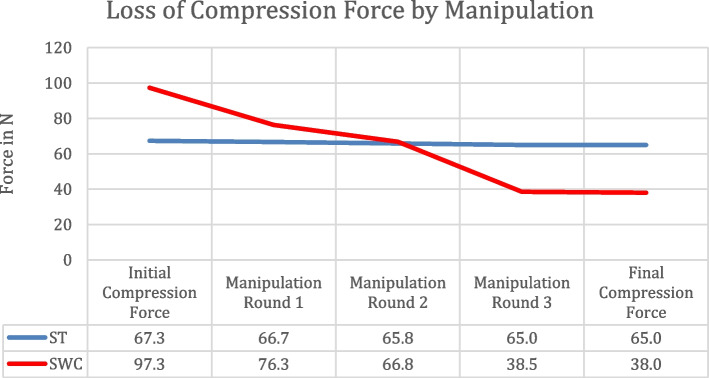
The effect of manipulation on a final wire cerclage construct is compared to a suture construct. Hence the suture construct is more robust against manipulation regarding loss of compression force

A compelling influence of manipulation on the compression force was detectable for all wire cerclage samples. In contrast to suture cerclages, the manipulation of the twisted lock resulted in a significant reduction of force of up to 61% from the initial values to final evaluation. In comparison, knot stack manipulation was more robust with showing only a limited influence of force reduction of around 3% in this sub-analysis (*p* < 0.001). (Fig. 5).

## Discussion

The main finding of the study is that all tested cerclage constructs have the capability to provide compression and retention stability to fulfill their task as an additive stabilizer for fracture treatment.

It has been shown that the increasing need for the strengthening support of the cerclage constructs mainly relies on the aging patient population with increasing numbers of periprosthetic fractures as one major indication [5, 9]. Furthermore, cerclages are nowadays not only used for fracture treatment but may also provide a protective character for primary arthroplasty implantation, especially in patients with reduced bone quality [10, 11]. Lastly, the application of cerclage constructs can help to manage reduction and serve as primary stabilization for complex fractures, outlining the variety of indications [12].

In accordance with the literature, this study shows comparable stability of modern high resistance suture tapes with metallic cerclages [8, 13] using a simplified fracture model. However, it has been shown that the biomechanical performance of a wire cerclage is affected significantly by the installation process [6, 7]. Hence, tensioning devices may help to increase the reproducibility and validity of the final constructs [10]. This study emphasizes that cerclage constructs should be used carefully, especially when tensioning devices will be used, as a substantial amount of force can be generated during cerclage implantation if not used properly.

One great fear when implementing multiple cerclages is the potential negative biologic impact on bone healing. Restriction of blood flow due to the surrounding character of cerclage wires has been stated to be a biological limitation and a cause of pseudarthrosis [1]. However, this could not be reproduced in simulation models [14]. The restricting effect may be influenced by the used material and initial force of installation. Within the groups, this study presents a difference in overall stiffness and force magnitude, offering a potential benefit for the less stiff non-metallic cerclage materials from a biological standpoint.

Due to the fact of different surface areas and material properties, it was hypothesized that.

differences could be detected by the interposition of muscular tissue between suture cerclage and bone compared to metallic cerclage construct. However, both materials were not influenced by the muscular interposition and provided equivalent stability regardless of soft tissue interposition. Be that as it may, it could be argued that the resulting muscular necrosis could lead to a secondary loosening of the constructs, and soft tissue interposition should therefore always be avoided.

Non-metallic cerclage materials like the used suture tapes outplay several disadvantages of their metallic precursors, like hardware prominence, risk of injury from sharp edges, potential allergies, and metallic ware [3, 4]. Additionally, M. Thomson et al. indicated that steel implants, especially cerclages, have the potential risk of increased corrosion due to friction. This may lead to encapsulation in connective tissue and increases the sensitivity to infections and loosening [15]. Hence, non-metallic cerclages can be used as material combination with a reduced risk profile.

Despite the aim of providing a reproducible and comparable testing scenario by reducing all variables down to the cerclage constructs and adding clinically relevant situations, some limitations still do exist in this study. First, all cerclages were installed under perfect conditions without restrictions in the field of view, neither for the passing process of the cerclage, which can be the most challenging part during surgery. Furthermore, only one force direction was conducted, which may not reflect the in-vivo forces of axial and torque strain completely. Additionally, the benefit or downside of cerclage stiffness cannot be proven and remains an unsolved clinical question, if applying cerclages too tight and too stiff may cause restriction of blood supply and therefore limit the healing process. Finally, the long-term survivorship of the materials, especially when running across sharp bone edges, remains unknown and cannot be answered. However, we did not see any signs of wear and tear of the suture tapes throughout the testing.

## Conclusion

All tested cerclage constructs produce reliable stability but differ in their resulting compression forces, in a simplified fracture model. Therefore, non-metallic cerclage alternatives can provide similar stability with less compression and stiffness to metallic cable constructs, but they may offer several advantages and could possibly provide future benefits. Especially, by offering more elasticity without losing overall stability, may offer a biologic benefit. Installing any cerclage constructs should be performed carefully, especially if poor bone quality is present, as the tightening process leads to high forces on the construct. Tensioning devices can help to provide more comparable constructs that are less prone to individual mistakes.

## Supplementary Information

**Additional file 1:** **Tab.1.** Additional data to accomplish the relevant findings described in themanuscript.

## Data Availability

The datasets generated and analyzed during the current study are not publicly available, but are available as de-identified data sheet from the corresponding author on reasonable request.

## Abbreviations

CC: Titanium Cable Cerclage
dSTC: Suture tape Cerclage, double looped
ST: Suture Tape
STC: Suture Tape Cerclage
sSTC: Suture Tape Cerclage, single looped
SWC: Steel Wire Cerclage
