# Supplementary material for: Cerclage performance analysis – a biomechanical comparison of different techniques and materials
Source: BMC Musculoskelet Disord. 2022 Dec 1;23:1037. doi: 10.1186/s12891-022-05983-6 (PMC9714204; doi:10.1186/s12891-022-05983-6)
Supplement: Supplementary file 1 — Additional file 1: Tab.1. Additional data to accomplish the relevant findings described in themanuscript. [file 12891_2022_5983_MOESM1_ESM.docx]

**Appendix: Cerclage performance analysis – A biomechanical comparison of different techniques and materials**

Tab. 1. Additional Data to accomplish the relevant findings described in the manuscript.
